# Caveolin and oxidative stress in cardiac pathology

**DOI:** 10.3389/fphys.2025.1550647

**Published:** 2025-02-18

**Authors:** Lauren Zadorozny, Jiayue Du, Neil Supanekar, Karthik Annamalai, Qing Yu, Meijing Wang

**Affiliations:** ^1^ Center for Surgical Sciences, Department of Surgery, Indiana University School of Medicine, Indianapolis, IN, United States; ^2^ Division of Cardiothoracic Surgery, Department of Surgery, Indiana University School of Medicine, Indianapolis, IN, United States

**Keywords:** reactive oxygen species, caveolae, heart disease, mitochondria, cardiomyopathy

## Abstract

Caveolins interact with signaling molecules within caveolae and subcellular membranes. Dysregulation of caveolin function and protein abundance contributes to cardiac pathophysiological processes, driving the development and progression of heart disease. Reactive oxygen species (ROS) play a critical role in maintaining cellular homeostasis and are key contributors to the pathophysiological mechanisms of cardiovascular disorders. Caveolins have been shown to modulate oxidative stress and regulate redox homeostasis. However, the specific roles of caveolins, particularly caveolin-1 and caveolin-3, in regulating ROS production during cardiac pathology remain unclear. This mini-review article highlights the correlation between caveolins and oxidative stress in maintaining cardiovascular health and modulating cardiac diseases, specifically in myocardial ischemia, heart failure, diabetes-induced metabolic cardiomyopathy, and septic cardiomyopathy. A deeper understanding of caveolin-mediated mechanisms may pave the way for innovative therapeutic approaches to treat cardiovascular diseases.

## Introduction

Caveolae, small (50–100 nm) invaginations in the plasma membrane, are found in various cell types within the cardiovascular system. They play crucial roles in regulating several cellular processes, including lipid and glucose metabolism, immune cell maturation, inflammatory responses, and nitric oxide synthase (NOS) signaling, among others (Panneerselvam et al., 2012; Schilling et al., 2018; Baev et al., 2022). Caveolins are essential for the formation of caveolae, acting as a signaling regulatory platform for receptor macromolecules and an integrative key factor in the cytoskeleton. Caveolins as receptor macromolecule and cellular skeleton in plasma membrane of human cells, and as modulators in inflammatory processes, have been summarized in other review articles (de Almeida, 2017; Thomas and Smart, 2008; Simon et al., 2020; Chidlow and Sessa, 2010; Correa et al., 2023; Brooks et al., 2023).

Oxidative stress, an imbalance between antioxidant defenses and the production of reactive oxygen species (ROS), plays a critical role in the pathophysiological process of many diseases. Despite extensive knowledge of the cardiovascular system, much remains to be understood about the regulation of redox homeostasis, specifically in the heart (Allemann et al., 2023; Goyal and Silversides, 2022; Afanas’ev, 2011). Excessive ROS impacts a range of proteins, altering mitochondrial bioenergetics and disrupting cellular function (Allemann et al., 2023). Caveolin has been shown to play a significant role in maintaining redox homeostasis, promoting cardiac function and preventing myocardial injury (An et al., 2024). In this mini-review article, we seek to update the contribution of caveolins to maintaining cellular redox homeostasis and regulating ROS production in health and pathophysiological conditions. We hope the exploration of caveolins, specifically in the heart, will pave the way for future studies aimed at developing potential therapeutical targets for cardiovascular diseases.

### Caveolin isoforms and their interrelationships

Three isoforms of caveolins have been identified: caveolin-1 (Cav-1), caveolin-2 (Cav-2), and caveolin-3 (Cav-3) (Gazzerro et al., 2011). They share similar three-dimensional structures and amino acid residue sequences, with Cav-1 playing the most dominant role in the formation of caveolae (Yang et al., 2021). Cav-1 and Cav-2 are widely expressed across various cell types, including adipocytes, endothelial cells, alveolar cells, and fibroblasts, whereas Cav-3 expression occurs specifically in muscle cells (cardiac, skeletal, and smooth muscle cells) (Schilling et al., 2018; Chen et al., 2017). In vertebrates, these three proteins are essential components of caveolae and are found in many types of cells, participating in various cellular structures such as the cell membrane, Golgi apparatus, endoplasmic reticulum, mitochondria, and vesicles (Fridolfsson et al., 2014). Each caveolin protein has a distinct expression pattern and function.

Cav-1 is highly characterized in terminally differentiated cells (Quest et al., 2008). It functions as a scaffolding protein and plays central roles in cholesterol transport, endocytosis, and signal transduction (Guo et al., 2011), as well as in regulating immune responses (Wang et al., 2006). In mitochondria, Cav-1 assists the regulation cholesterol through mitochondrial-associated membranes (MAMs). On the membrane domains of MAMs, the lipid raft is rich in cholesterol and acts as a temporary signaling platform (Bernal et al., 2023), where Cav-1 can further be used as a binding site to control different mitochondrial metabolic processes. Of note, due to its powerful hydrophobic scaffolding domains, Cav-1 not only forms caveolae and large homo-oligomers but also constructs hetero-oligomers with the other two caveolins within cardiomyocytes (Benzoni et al., 2024), which allows for the regulation of angiogenesis, vascular permeability, and transcytosis (Frank et al., 2003).

Meanwhile, the expression of Cav-1 and Cav-2 is interdependent. Cav-2 levels are rarely observed in Cav-1 knockout (KO) mice (Razani et al., 2001; Drab et al., 2001), and the absence of Cav-2 results in reduction of Cav-1 (Razani et al., 2002). Cav-1 stabilizes Cav-2, facilitating redistribution to caveolae membranes (Parolini et al., 1999). Without Cav-1, Cav-2 localizes to Golgi complex, where it becomes a target for degradation (Parolini et al., 1999). Furthermore, co-expression of Cav-1 and Cav-2 can lead to the formation of caveolae deeper within fibroblasts (Fujimoto et al., 2000). Additionally, opposing effects of Cav-1 and Cav-2 have been noted in tumor-induced angiogenesis (Capozza et al., 2012; Woodman et al., 2003), suggesting that Cav-2 is not only a partner of Cav-1 in caveolae formation but also assists in fine-tuning basic cellular processes in a counterbalancing manner (de Almeida, 2017).

A database search identified Cav-3 as a homolog of Cav-1, sharing 65% sequence identity and 85% similarity with Cav-1 (Tang et al., 1996). Cav-3 serves similar roles as Cav-1 in aiding caveolae formation and regulating the activities of various proteins (Hagiwara et al., 2000). However, Cav-3 is located within the sarcolemma of the muscle fiber membrane (Lory et al., 2020) and present in T-tubules (transverse tubules), promoting development, sustainment, and repairment processes in the muscle cells. Cav-3 is also co-localized with dystrophin (Song et al., 1996) to support the production and expression of myoblasts and myotubes (Galbiati et al., 1999). As an essential structural component of muscular caveolae, Cav-3 mutations disrupt its interaction with Cav-1, leading to channel dysfunction in the heart (Benzoni et al., 2024).

Each type of caveolins exhibits both similarities and differences in function, expression, and other characteristics, contributing to the formation of caveolae within the cells and regulating various cellular processes, such as endocytosis, exocytosis, signaling molecules, mechanoprotection, and lipid regulation (D'Alessio, 2023).

### Different isoforms of caveolin in regulating ROS production

#### Caveolin-1

Cav-1 maintains the integrity and function of mitochondria when cells are confronted with free radicals by quality control of the mitochondrial proteases (Volonte et al., 2016). By interacting with the fusion machinery of mitochondria, Cav-1 prevents damage and accumulation of ROS. Cav-1 deficiency results in the augmentation of cholesterol, increasing the risk of mitochondrial failure, exacerbating oxidative stress, and promoting apoptosis (Bosch et al., 2011).

Accumulating evidence suggests that Cav-1’s roles in modulating oxidative stress are cell type-dependent. In some cells, Cav-1 exerts protective anti-oxidative effects. For instance, the downregulation of Cav-1 has been linked to mitochondrial enzymatic dysfunction and increased ROS production in stromal cells (Nwosu et al., 2016). Cav-1 prevents endothelial dysfunction by inhibiting ATP7A ubiquitination and proteasomal degradation, thereby increasing SOD3 activity to reduce vascular oxidative stress (Sudhahar et al., 2020). Additionally, upregulation of Cav-1 protected lung cancer cells from excessive ROS-induced cell death (Wu et al., 2023). By contrast, Cav-1 has been reported to suppress nuclear factor erythroid 2-related factor 2 transcriptional activity, therefore downregulating antioxidant enzymes such as GCLC and HO-1 in pneumocytes (Li et al., 2012). Furthermore, bronchial epithelial cells overexpressing Cav-1, exhibited increased susceptibility to H_2_O_2_-induced cell death (Xu et al., 2020). These studies thus highlight the distinct roles of Cav-1 in regulating oxidative stress across different cell types.

It is unclear whether Cav-1’s effect on oxidative stress is also specific to disease type. There are several studies demonstrating that Cav-1 affects ROS differently in various diseases. For example, in carbon tetrachloride-induced hepatic damage, Cav-1KO mice exhibited exaggerated liver injury and increased total ROS levels compared with wild-type mice. Cav-1 deficiency further intensified oxidative stress responses, including reduced antioxidant defense (superoxide dismutase activity and glutathione [GSH] levels), along with increased malondialdehyde and O_2_^−^ levels (Ji et al., 2018). Conversely, in acetaminophen-induced hepatotoxicity, Cav-1KO mice showed significantly attenuated liver damage with altered antioxidants (Gardner et al., 2010). Meanwhile, in neuropathology, Cav-1 protected astrocytes from oxidative stress (Yun et al., 2011). In addition, Cav-1 reduced disruption of the blood brain barrier via decreasing ROS production and inhibiting the activity of matrix metalloproteases during ischemia stroke (Huang et al., 2018). In nephropathy, Cav-1 was also identified as a protector to mitigate damage in E11 murine kidney podocytes by stimulating antioxidant enzymes, thus decreasing oxidative stress induced by H_2_O_2_ (Chen et al., 2017). By contrast, oxidative stresses foster the translocation of Cav-1, generating the senescence of fibroblasts in lungs (Volonte et al., 2016; Volonte and Galbiati, 2009).

#### Caveolin-2

Interestingly, Cav-2 appears to exert opposing effects compared to Cav-1. For example, Cav-2 KO mice are more sensitive to LPS-caused tissue damage, while delayed mortality is observed in LPS-treated Cav-1 KO mice (de Almeida et al., 2011). This difference can be attributed to increased induced nitric oxide synthase (NOS) expression and nitric oxide (NO) production in endotoxin-stimulated Cav-2 KO mice, but not in Cav-1 KO animals. Notably, the role of Cav-2 in regulating cellular redox homeostasis is far less studied than Cav-1, with limited research on Cav-2’s role in ROS production or metabolic regulation (Sowa, 2012).

#### Caveolin-3

In muscle cells, Cav-3 dissociates from caveolae to localize in the cytosol or mitochondrial membrane, where it enhances respiratory capacity and mitigates oxidative stress (Fridolfsson et al., 2012). A deficiency of Cav-3 in the mitochondria disrupts cellular function, particularly to exacerbate oxidative stress. Excessive ROS formation reduces mitochondrial membrane potential and biogenesis, leading to mitochondrial dysfunction and apoptosis (Chen et al., 2023). Cav-3 works to prevent this by regulating ion channels that interact with the electron transport chain (ETC.) on mitochondria to slow ROS release (Zhao et al., 2019). Such evidence has promoted the significant importance of Cav3 on mitochondria and its response to stress adaptation.

In healthy skeletal muscle cells, ROS production is balanced by antioxidant enzymes. However, ROS generation can exceed the cell’s antioxidant ability when there is an excess of mitochondrial fuel supply and/or reduced expression or activity of antioxidant enzymes (Zorov et al., 2014). Mutations of the Cav-3 gene lead to Cav-3 protein deficiency, disrupting mitochondrial homeostasis and causing ROS production imbalance, leading to muscle development and stability defects. Additionally, mutations of the muscle-specific proteins: Cav-3, dysferlin, and mitsugumin 53 (MG53) can disrupt the repair process, resulting in the progression of muscular dystrophies (Cai et al., 2009a; Bansal et al., 2003). MG53, an oxidation signaler, works with Cav-3 to promote the repair of injury sites on cellular membranes (Cai et al., 2009b) through the improvement of dysferlin trafficking (Cai et al., 2009a). Mutations in Cav-3 disrupt the functional interaction between MG53 and dysferlin (Hernández-Deviez et al., 2006). Thus, mutations in Cav-3 are linked to various muscular dystrophies (Shah et al., 2020; Bouchard and Tremblay, 2023).

Collectively, Cav-1 and Cav-3 influence processes in specialized membrane domains, such as those on mitochondria, plasma membranes, and T-tubules, to ensure proper cell function. Alterations in these proteins can lead to ROS alterations at various levels, further activating distinct signaling pathways and biological processes within organisms (Schieber and Chandel, 2014).

### Anti-oxidative stress of caveolin in cardiac pathogenesis

**FIGURE 1:**
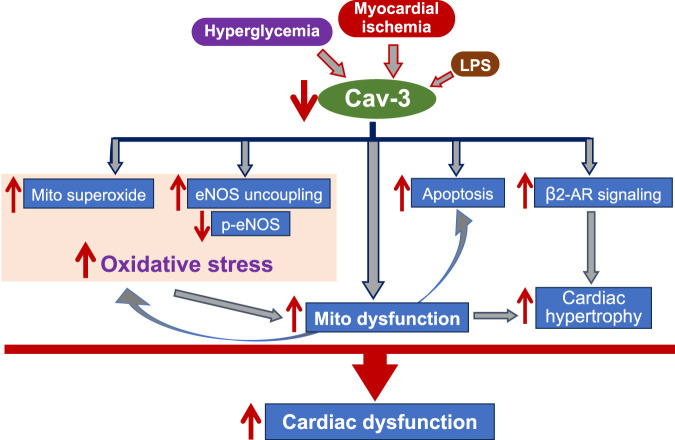
The flow chart indicates the mechanisms underlying Cav-3-mediated cardiac protection. Cav-3 conveys protective effects on cardiac function during myocardial ischemia, progression of heart failure, hyperglycemia, and endotoxemia, through anti-oxidative stress, regulating mitochondrial function, anti-apoptosis, and modulation of β2-AR signaling. Mito: mitochondria; LPS: lipopolysaccharide; eNOS: endothelial nitric oxide synthase; p-: phosphor; NO: nitride oxide; β2-AR: β2-adrengergic receptor.

Cardiovascular diseases often arise from disruptions in the signaling processes of ROS and reactive nitrogen species (RNS) (Afanas’ev, 2011). Given that the role of Cav-2 in cardiovascular disease remains understudied, this mini-review mainly focuses on discussing the presence and function of Cav-1 (Table 1) and Cav-3 (Figure 1) in influencing oxidative stress relevant to the pathogenesis of various heart diseases.

**TABLE 1:** Caveolin-1 in cardiac oxidative stress.

Animal/Tissue	Signaling pathway/mechanism	Cardiac phenotype and outcome	Cells involved in injury	Disease model	References
*Cav1* ^*−/−*^ mice	Cav-1KO → ↓β-AR → ↓ cardiac cAMP → ↓ p-PKA	↓LVEF, LVFS ↑LVEDP ↓Survival Rate	-	MI	Jasmin et al. (2011)
*24-month-old rats*	Dissociation of Cav-1 from caveolae → ↑Cytosolic Cav-1/NOS3 complexes	↑LVEDP	ECs and SMCs	Heart Failure	Ratajczak et al. (2003)
*Cav1* ^*−/−*^ mice	Cav-1KO → ↑eNOS activity → ↑Systemic NO_x,_ ↑O_2_^−^	↓LVDP, dP/dt_max_, dP/dt_min_ ↑LVEDP	-	Heart Failure	Wunderlich et al. (2008)
*Cav1* ^*−/−*^ *and Cav1* ^*−/−*^ */eNOS* ^*−/−*^ *mice*	↓Cav-1 → vascular eNOS dysfunction → ↑ROS	↓LVDP, dp/dt_max_, dp/dt_min_ ↓stroke volume, cardiac output ↑Aortic diameter, SP, diastolic pressure ↑Wet heart weight, LV posterior wall thickness	-	Heart Failure	Ebner et al. (2017)
*Cav1* ^*−/−*^ mice	Chronic hypoxia → ↑ eNOS uncoupling, ↑p-eNOS (S1176) → ↑Superoxide, ↓NO→ ↑Oxidative/nitrosative stress in RV	↓RVSP, cardiac output ↑RV hypertrophy and RV interstitial fibrosis	-	Right Heart Failure	Cruz et al. (2012)
*Cav1* ^*−/−*^ mice	Cav-1KO → ↑eNOS activity → ↑NO in vasculature Cav-1KO → ↑p-Akt and p-p42/44 → hyperproliferation, ↑collagen synthesis	LV and RV cardiac hypertrophy ↑ Coronary artery diameter and wall thicknesses ↑ RVSP, RVDP, RVEDP ↓Systemic arterial pressure	ECs	Heart Failure	Murata et al. (2007)
*Cav1* ^*+/+*^ and *Cav1* ^*−/−*^ diabetic mice	Cav-1KO → ↓IκBα → ↑NF-κB → ↑ANF, BNP, FN, ICAM-1 and TGF-β1	Enlarged heart ↑Cardiac collagen fiber	CMs H9C2	DCM	Gong et al. (2023)
Human skeletal muscle biopsies	↓Cav-1 → ↓eNOS, ↑p-eNOS →↓insulin uptake; ↑Cav-1 → stabilize eNOS	-	ECs	Type 2 diabetes	Chen et al. (2018)
*Trpc1* ^−/−^ mice and Cavtratin -treated mice	LPS → ↑TRPC1 → ↑intracellular Ca^2+^ release → ↓Cav-1 → ↑ apoptosis and autophagy	↑LVEF, LVFS ↑Survival Rate	-	SCM	Tian et al. (2022)
*Cav1* ^*−/−*^ mice and blood vessels	Cav-1KO →↓ATP7A → ↓SOD3 activity → ↑O_2_^−^→endothelial dysfunction	Impaired endothelium-dependent vasorelaxation	Vascular cells, Fibroblast	-	Sudhahar et al. (2020)
*eCav-1* ^*−/−*^ mice and isolated hearts	Cav-1KO → ↑eNOS activity, ↓p-eNOS (Thr495) → ↑NO → ↑ cardiac nitrative stress	Cardiac hypertrophy ↑ Baseline coronary flow	Vascular cells, ECs	-	Saito et al. (2018)

eCav-1-KO, endothelium-specific Cav-1-knockout; eNOS, endothelial Nitric Oxide Synthase; TRPC1, Transient receptor potential canonical channel1; LV, Left Ventricular; EF, Ejection Fraction; FS, Fractional Shortening; EDP, End Diastolic Pressure; DP, Developed Pressure; d*P*/d*t*_max/min_, maximal/minimal first derivative of the LV pressure over time; RV, Right Ventricular; SP, Systolic pressure; β-AR, β-adrenergic receptor; cAMP, cyclic adenosine 3′,5′-monophosphate; PKA, Protein kinase A; NO_x_, nitrate and nitrite; IκBα, Inhibitor α of NF-κB; ANF, Atrial Natriuretic Factor; BNP, Brain Natriuretic Peptide; FN, fibronectin; ICAM-1, Intercellular adhesion molecule-1; TGF-β1, transforming growth factor-β1; iNOS, inducible NO synthase; ECs, Endothelial Cells; SMCs, Smooth Muscle Cells; CM, cardiomyocytes; MI, Myocardial Ischemia; DCM, Diabetic Cardiomyopathy; SCM, Septic Cardiomyopathy.

#### Myocardial ischemia/reperfusion (I/R)

Myocardial ischemia (MI) arises during occlusion of coronary vessels or cardiac surgical operations (Cassar et al., 2009). Due to their vast energy consumption for sustaining heartbeat, cardiomyocytes are vulnerable to ischemia and subjected to injuries on the subcellular and cellular levels. Among these, damaged mitochondria during MI manifest increased membrane permeability, excessive ROS production, calcium overload, and release of intrinsic apoptotic proteins, collectively contributing to cardiomyocyte death (Kantrow and Piantadosi, 1997; Suliman and Piantadosi, 2016; Taylor et al., 1995; Kalogeris et al., 2012). The restoration of blood flow to the heart (reperfusion) remains the treatment of choice for heart ischemia. However, surges of ROS generation by reperfusion, which cannot be checked by dysfunctional mitochondria, aggravate cardiomyocyte impairment. Therefore, controlling I/R-induced oxidative stress is important to ameliorate myocardial damage following I/R injury.

Emerging evidence found Cav-1 and caveolar domains as a source of protection against pathogenesis for MI (Jasmin et al., 2011). All cardiac cell types including cardiomyocytes express Cav-1 (Chow et al., 2007; Patel et al., 2007; Robenek et al., 2008). Cav-1 was dissociated from caveolae to the cytosol in rat hearts after myocardial infarction (Ratajczak et al., 2003), which increased cytosolic Cav-1/endothelial NOS (eNOS) complexes, and subsequently, decreased the production of NO, a molecule to limit deleterious effects of ROS (Ratajczak et al., 2003). Cav-1KO mice displayed damaged cardiac function and reduced survival following MI, likely due to microvascular hyperpermeability and reduced vascular tone to eNOS (Razani et al., 2001; Jasmin et al., 2011; Schubert et al., 2002). In fact, the Cav-1 scaffolding domain has been shown to directly interact with eNOS to inhibit eNOS activity (Garcia-Cardena et al., 1997; Garcia-Cardena et al., 1996).

The link between Cav3 and cardiac protection has emerged from accumulated studies (Tsutsumi et al., 2008; Horikawa et al., 2008; Tsutsumi et al., 2010; Tsutsumi et al., 2014; See Hoe et al., 2014). Notably, regulating caveolin in mitochondria is important to cell survival during stress (Fridolfsson et al., 2014; Fridolfsson et al., 2012; Schilling and Patel, 2016). The presence of Cav-3 in cardiomyocyte mitochondria has been shown to improve respiration and calcium tolerance and reduce ROS production (Fridolfsson et al., 2012). Cav-3 overexpression decreases superoxide generation, while its deficiency increases superoxide production (Guo et al., 2024). ROS, a main cause of reperfusion injury (Sanderson et al., 2013), is surged by the mitochondria-activated redox signaling (Granger and Kvietys, 2015). Cav-3 can modulate mitochondrial function and mitigate oxidative damage, conveying cardioprotective effects. Indeed, Cav-3 overexpression (Cav-3OE) decreased ROS generation in cardiomyocytes subjected to simulated I/R, whereas cardiomyocytes from Cav-3KO mouse hearts have shown increased superoxide production (Fridolfsson et al., 2012). In line with it, more tightly coupled Complex I was observed in mitochondria with Cav-3 overexpression, leading to less leakage of mitochondrial ROS compared to those from Cav-3KO mouse hearts (Fridolfsson et al., 2012). An additional study has also reported that ischemic preconditioning-further increased mitochondrial Cav-3 expression and eNOS provided greater cardiac protection in the ischemic heart (Sun et al., 2015). Notably, Cav-3 could also protect cardiomyocytes from TNF- or hypoxia-induced cell death through its anti-apoptotic effect (Carotenuto et al., 2013; Zhou et al., 2018).

#### Heart failure

Heart failure occurs when the cardiac muscles cannot pump enough blood to meet the body’s needs (Ziaeian and Fonarow, 2016). A key pathophysiological factor associated with the development and progression of heart failure is oxidative stress. An increase in ROS impairs the electrophysiology and the contractile mechanism of cardiomyocytes (Takimoto and Kass, 2007). The surge in ROS directly weakens contractile performance by altering proteins in excitation-contraction (EC) coupling, leading to a Ca2+ overload which causes more stress onto the heart and mechanical abnormalities (Luo and Anderson, 2013). Production of ROS within the mitochondria via the, ETC., further induces massive quantities of superoxide production to trigger Ca2+ release to alter contractions (van der Pol et al., 2019).

Cav-1 is beneficial for limiting the development of heart failure. Cav-1 governs cardiac activity by regulating eNOS and synthesizing NO in the heart (Tian et al., 2020). In Cav-1KO mice, severe cardiomyopathy with impaired pump function was observed due to negatively regulated eNOS that led to constitutive hyperactivation of the NO pathway (Wunderlich et al., 2008; Ebner et al., 2017). Additionally, eNOS uncoupling in Cav-1KO mice increased oxidative and nitrosative stress, thereby altering the response of the right ventricle to pressure overload and promoting the development of right heart failure (Cruz et al., 2012; Shu and Jin, 2023). Importantly, the reconstitution of Cav-1 (Wunderlich et al., 2008), particular the endothelium-specific re-expression of Cav-1 (Murata et al., 2007), reversed cardiac hypertrophy in global Cav-1KO mice.

During heart failure, t-tubules play a key role in preserving the ejection fraction and mitigating damage to the heart (Setterberg et al., 2021). Abundant with Cav-3, t-tubules facilitate the rapid conduction of electrical signals and enhance communication within the sarcoplasmic reticulum (Huxley and Taylor, 1958). This communication, occurring through EC coupling, ensures the proper calibration and release of Ca2+, thereby supporting efficient muscle contractions. Loss of Cav-3 has resulted in significant cardiac hypertrophy and reduced myocardial function (Woodman et al., 2002). Cav-3KO mice expressed cardiac dysfunction associated with t-tubule reorganization and decreased L-type calcium channel (LTCC) current density (Bryant et al., 2018). Additionally, reduced Cav-3 level in the heart has been shown to increase myocyte dis-contractility and the progression of heart failure through enhanced susceptibility to ventricular arrhythmia (Markandeya et al., 2023) or β_2_AR signaling (Barbagallo et al., 2016). More importantly, mitochondria are the primary source of energy for the heart, and their dysfunction contributes to the development of heart failure. Cav-3 has been shown to provide mitochondrial protection in the heart. In mouse hearts lacking Cav-3, increased superoxide signals and enhanced ROS production were observed, contributing to the progression of cardiac pathology (Fridolfsson et al., 2012).

Notably, both Cav-1 and Cav-3 have been observed in rat and mouse cardiomyocytes (Head et al., 2006). The mice with deficient in both Cav-1 and Cav-3 develop more severe cardiomyopathy compared to the mice with deficient in either Cav-1 or Cav-3 (Park et al., 2002). These findings suggest that modulating cardiac Cav-1 or Cav-3 expression may serve as a therapeutic strategy to mitigate cardiac hypertrophy and heart failure.

#### Diabetic cardiomyopathy

A major complication of diabetes is diabetic cardiomyopathy (DCM), characterized by left/bi-ventricular dilation, impaired contraction, cardiac remodeling, and dysfunction (Schultheiss et al., 2019). The onset of DCM begins shortly after diabetes establishment, progressing to ventricular dysfunction and cardiac hypertrophy (Oktay et al., 2024). This causes a loss of the glucose transporter type 4 (GLUT-4) in skeletal and cardiac muscle tissues, resulting in a reduction of glucose and ATP in cells (Murfitt et al., 2015). Studies revealed that the morphological localization of GLUT-4 in caveolin after insulin stimulation caused a shift in the cell’s structure and hindered various vital cellular mechanisms (Karlsson et al., 2002), like muscle contractibility. Evidence has found caveolae are essential for insulin signaling pathways, specifically the phosphorylation of Cav-1, allowing for insulin receptor activity and protection from degradation pathways to be activated (Mastick et al., 1995). Inhibition of insulin sensors increases the amount of glucose in the body, further leading to hyperglycemia, a hallmark characteristic of diabetes. Investigations revealed more severe cardiac injury in Cav-1KO mice, with Cav-1 deficiency leading to increased failure of the insulin signaling pathway, resulting in enhanced activation of nuclear factor kappa-B (NF-kB) signaling (Gong et al., 2023).

In DCM, hyperglycemia increases mitochondrial ROS production and calcium ion levels while impairing the eNOS pathway (Li et al., 2023). eNOS-regulated NO production plays a critical role in endothelial cell function, controlling vascular tone, blood flow, and circulation in the heart (Tran et al., 2022). Evidence highlights the interplay of caveolins with eNOS being essential for NO production, thus maintaining oxidative balance and mitigating stress (Wang et al., 2017). Dysregulated eNOS signaling is pivotal in the progression of DCM. Reduced expression of Cav-1 and eNOS has been observed in endothelial cells from human muscle biopsies with type 2 diabetes (Chen et al., 2018). Additionally, in human aortic endothelial cells exposed to high glucose, increased eNOS-Cav-1 interaction and reduced NO availability have been linked to mitochondrial oxidative stress, contributing to diabetic cardiovascular complications (Paneni et al., 2015). It is worth noting that a key mechanism in the development of DCM is the impairment of Cav-3/eNOS complex formation. Cav-3 interacts with eNOS signaling at the mitochondria, facilitating NO release to counteract excessive ROS production (Yu et al., 2011). Additionally, reduced cardiac Cav-3/eNOS signaling has been found in diabetic rats with DCM (Su et al., 2016), whereas antioxidant treatment using N-acetylcysteine restored cardiac Cav-3/eNOS signaling (Su et al., 2016). These findings highlight a complex interplay between eNOS, Cav-1, and Cav-3 in regulating the development and progression of DCM.

#### Septic cardiomyopathy

Septic cardiomyopathy (SCM) is a form of non-ischemic cardiac dysfunction (Boissier and Aissaoui, 2022), characterized by global biventricular impairment, reduced contractility, left ventricular dilation, and decreased responsiveness to fluid resuscitation (L'Heureux et al., 2020). While the pathophysiology of SCM is not fully understood, it is believed to be multifactorial, involving mitochondrial dysfunction, oxidative stress, pathogen-associated molecular patterns (PAMPs), damage-associated molecular patterns (DAMPs), NO release, and metabolic disturbances (Wenceslau et al., 2014; Nakahira et al., 2015). Recent studies have shown that severe sepsis induces mitochondrial structural abnormalities, impairing their function and increasing ROS production in the heart (Lira Chavez et al., 2023; Van Remmen and Richardson, 2001). These disruptions impact NO release, alter preload and afterload, downregulate beta-adrenergic receptors, and reduce myofilament sensitivity to calcium ions (Hollenberg and Singer, 2021) - all weakening cardiac function and increasing susceptibility to sepsis.

Evidence from both preclinical and clinical studies has demonstrated that excessive ROS is associated with several stages of sepsis. While Cav-1 plays a role in sepsis by regulating membrane traffic and intracellular signaling pathways (Lannes-Costa et al., 2022), Cav-1 directly modulates ROS production via NADPH oxidase (Michell et al., 2021; Cai et al., 2014), as NADPH oxidase subunits have been shown to localize within Cav-1-containing caveolae across various cell types (Ushio-Fukai, 2006). Through its regulation of ROS, Cav-1 influences immune cell function and modulates apoptosis and autophagy during sepsis (Lannes-Costa et al., 2022). Additionally, decreased Cav-1 expression in LPS-challenged mice resulted in increased myocardial apoptosis and autophagy (Tian et al., 2022). Increased myocardial damage (↑pseudocysts, myonecrosis and inflammation in the heart) with reduced survival was observed in Cav-1KO mice underwent acute infection, likely attributed to decreased NO production and promoted immune dysfunction (Medina et al., 2007). Cav-1 overexpression reduced eNOS activity, whereas LPS–induced pulmonary hyperpermeability and edema were observed in Cav-1KO mice, attributed to increased eNOS activity (Chen et al., 2018; Garrean et al., 2006). A recent paper provides a comprehensive review of Cav-1’s role in sepsis (Lannes-Costa et al., 2022).

Unlike myocardial ischemia and heart failure, severe sepsis significantly increased Cav-3 expression in the heart, which was associated with dysregulated L-type calcium channels (Rattis et al., 2021). Treatment with a calcium channel inhibitor decreased Cav-3 levels and improved survival rates in septic mice (Rattis et al., 2021). Conversely, selective inhibition of PKCβ2 restored LPS-impaired Cav-3/eNOS signaling, thereby mitigating autophagy and LPS-induced myocardial injury (Yang et al., 2021). These contrasting findings suggest that cardiac response to sepsis may vary depending on the type of septic stimulation, and the precise mechanisms underlying Cav-3’s role in sepsis require further exploration. Notably, these studies did not specifically address the role of mitochondrial Cav-3 in septic cardiomyopathy. It also remains unknown whether Cav-3 can dissociate from membranes or caveolae, and if so, whether the dissociated Cav-3 retains functional activity in sepsis.

## Conclusion and future prospect

Growing evidence suggests that caveolin expression plays a crucial role in protecting cardiac function and preventing myocardial damage. In this mini-review article, we summarized the protective effects of Cav-1 (Table 1) and Cav-3 (Figure 1) on damaged hearts through the anti-oxidative stress mechanism. The complex ways by which caveolins regulate ROS production may offer valuable insights into potential therapeutic approaches (Schilling et al., 2018; Fridolfsson et al., 2014). For example, targeting the eNOS signaling complex in response to dysregulated redox balance in caveolae via caveolins could be beneficial. NO contributes to regulating ROS metabolism and hypertrophic signaling in cardiomyocytes. Consequently, strategies aimed at modulating Cav-1 and Cav-3 interactions with eNOS and the resulting NO release hold potential for cardiac protection (Baev et al., 2022).

Additionally, the caveolin scaffolding domain (CSD) plays an important role in regulating protein - protein interactions, mediating the interplay between caveolins and various signaling proteins (Panneerselvam et al., 2012). Studies have shown that using the CSD peptide of Cav-1 protected the heart from I/R injury and improved cardiac functional recovery (Young et al., 2001), highlighting its therapeutic potential. Despite these promising findings on Cav-1 and Cav-3, the complex signaling networks involved in their regulation remain elusive. Further research should focus on using more precise strategies to elucidate the roles of caveolins in cardiac cell biology and the mechanisms by which caveolins contribute to the pathogenesis of the cardiovascular diseases. The insights gained from this work will help advance the development of therapeutic approaches targeting the modulation of caveolin expression and function for the treatment of cardiovascular disease.
